# Phosphoproteomics analysis of male and female *Schistosoma mekongi* adult worms

**DOI:** 10.1038/s41598-019-46456-6

**Published:** 2019-07-10

**Authors:** Nattapon Simanon, Poom Adisakwattana, Tipparat Thiangtrongjit, Yanin Limpanont, Phiraphol Chusongsang, Yupa Chusongsang, Songtham Anuntakarun, Sunchai Payungporn, Sumate Ampawong, Onrapak Reamtong

**Affiliations:** 1 Department of Molecular Tropical Medicine and Genetics, Faculty of Tropical Medicine, Mahidol University, Bangkok, 10400 Thailand; 2 Department of Helminthology, Faculty of Tropical Medicine, Mahidol University, Bangkok, 10400 Thailand; 3 Department of Social and Environmental Medicine, Faculty of Tropical Medicine, Mahidol University, Bangkok, 10400 Thailand; 4 Department of Biochemistry, Faculty of Medicine, Chulalongkorn University, Bangkok, 10330 Thailand; 5 Department of Tropical Pathology, Faculty of Tropical Medicine, Mahidol University, Bangkok, 10400 Thailand

**Keywords:** Gene ontology, Phosphorylation

## Abstract

*Schistosoma mekongi* is one of the major causative agents of human schistosomiasis in Southeast Asia. Praziquantel is now the only drug available for treatment and there are serious concerns about parasite resistance to it. Therefore, a dataset of schistosome targets is necessary for drug development. Phosphorylation regulates signalling pathways to control cellular processes that are important for the parasite’s growth and reproduction. Inhibition of key phosphoproteins may reduce the severity of schistosomiasis. In this research, we studied the phosphoproteomes of *S*. *mekongi* male and female adult worms by using computational and experimental approaches. Using a phosphoproteomics approach, we determined that 88 and 44 phosphoproteins were male- and female-biased, respectively. Immunohistochemistry using anti-phosphoserine antibodies demonstrated phosphorylation on the tegument and muscle of male *S*. *mekongi* worms and on the vitelline gland and gastrointestinal tract of female worms. This research revealed *S*. *mekongi* sex-dependent phosphoproteins. Our findings provide a better understanding of the role of phosphorylation in *S*. *mekongi* and could be integrated with information from other *Schistosoma* species to facilitate drug and vaccine development.

## Introduction

Human schistosomiasis is a helminthic infectious disease caused by blood-dwelling parasites of the genus *Schistosoma*; *S*. *haematobium*, *S*. *mansoni*, *S*. *japonicum*, *S*. *intercalatum*, and *S*. *mekongi* are known to infect humans. The first case of Mekong schistosomiasis was reported in Southeast Asia in 1957^1^. *Neotricula aperta* is a snail-intermediate host of *S*. *mekongi*. The habitat of this snail is the Mekong River basin. To date, approximately 60,000 and 80,000 people in Laos and Cambodia, respectively, are estimated to be at risk of infection with *S*. *mekongi*^2^. Similarly to other species, *S*. *mekongi* resides in the host’s mesenteric vasculature^3,4^. Until now, no successful vaccine has been schistosomiasis. The tolerance or resistance to praziquantel (PZQ) developed for any schistosomiasis and PZQ remains the only drug used for schistosomiasis treatment^5–8^. Male-female pairing is important for female worm development and reproduction, and thus egg production and fecundity, which is the cause of pathogenesis^9,10^. Inhibition of *Schistosoma* maturation and egg production may lead to reduced pathogenesis and transmission to the host. Therefore, basic knowledge of the molecular biology of the species and a library of drug target and vaccine candidates are crucial for control of schistosomiasis.

A key reversible posttranslational modification, phosphorylation, drives the signalling pathways of several cellular processes^11,12^. Phosphorylation is dynamically controlled by networks of kinases and phosphatases. Addition of phosphate groups can alter protein activity, stability, localization, and interactions, and abnormal phosphorylation is involved in many diseases such as cancer, diabetes, and neurodegeneration^13^. Recent advances in bioinformatics and proteomics methods make it is possible to identify large-scale protein phosphorylation in many organisms. Phosphoproteomes of not only humans but also parasites have been revealed. In *S*. *japonicum*, 92 phosphoproteins were identified using mass spectrometry^14^. Heat shock protein 90 (Hsp90) was phosphorylated and the parasite was killed after Hsp90 inhibitor treatment of *in vitro*-cultured *S*. *japonicum*. Therefore, Hsp90 is a potential therapeutic target for *S*. *japonicum* infection^14^. *S*. *mansoni* adult male worms exposed to human tumour necrosis factor-α (hTNF-α) showed statistically significant increases in phosphorylation of proteins involved in glycolysis, galactose metabolism, urea cycle, and aldehyde metabolism^15^. The finding suggests that hTNF-α plays a role in crosstalk between host and pathogen through phosphorylation. Few studies have been published on *Schistosoma* phosphoproteomes, especially that of *S*. *mekongi*.

In this study, we studied the phosphorylation of *S*. *mekongi* male and female adult worms by bioinformatic prediction. In addition, we explored phosphoproteins that were differential between the sexes by using two-dimensional gel electrophoresis (2-DE) followed by mass spectrometry (MS). We performed immunohistochemistry on tissues of *S*. *mekongi* male and female adult worms to reveal the dominant phosphorylated organs. This study provides information on *S*. *mekongi* sex-dependent phosphoproteins, which may facilitate the development of agents to block parasite development, maturation, male-female pairing, and egg production. The information presented here may enhance our understanding of human-infecting schistosomes to improve *Schistosoma* prevention and control.

## Results

### Prediction of *S*. *mekongi* phosphoproteins

**Figure 1:**
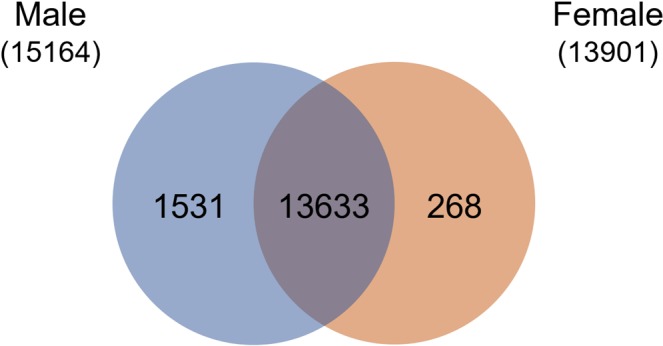
Venn diagram of predicted phosphoproteins using NetPhos 3.1 server of *S*. *mekongi* male and female worms.

According to *S*. *mekongi* transcriptomic data, 20,795 annotated proteins were subjected to prediction of potential phosphorylation sites using the NetPhos 3.1 server. In total, 15,432 proteins contained at least one significant phosphorylation site on serine, threonine, or tyrosine residues (Supplementary Datasets 1 and 2). Potential phosphoproteins in male and female worms totalled 15,164 and 13,901 proteins, respectively (Fig. 1), with 13,633 phosphoproteins being expressed in both sexes. Overall, 1,531 and 268 phosphoproteins were predicted to be male- and female-biased, respectively (Supplementary Datasets 1 and 2).

### *S*. *mekongi* phosphoproteomics

**Figure 2:**
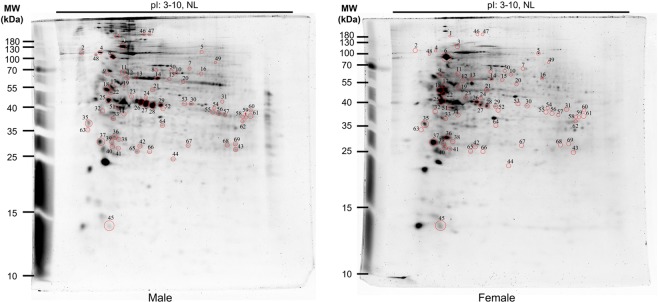
2-DEs of male (**a**) and female (**b**) adult worm phosphoproteins visualized by Pro-Q Diamond staining.

**Figure 3:**
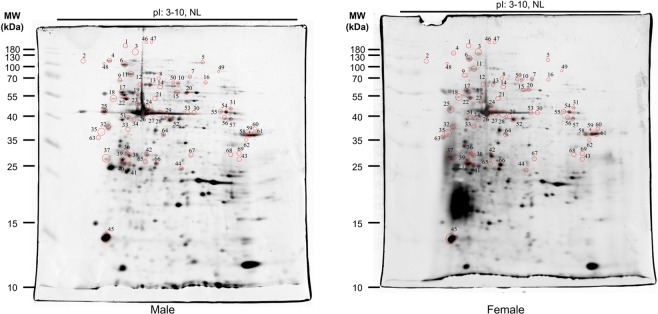
2-DEs of male (**a**) and female (**b**) adult worm proteins visualized by silver staining.

Although bioinformatics can predict phosphorylation sites in eukaryotic proteins, it does not yield definitive or quantitative information. To that end, we performed relative quantification of phosphoproteomes of *S. mekongi* male and female adult worms using traditional 2-DE. The gels were stained with a fluorescent dye that specifically binds to phosphoproteins (Pro-Q Diamond staining). Gel images were scanned and spot intensities quantified using ImageMaster software (Fig. 2). A total of 450 and 429 spots were presented on the Pro-Q Diamond stained gels of male and female worms, respectively. Of these, 50 spots were male-biased and 19 were female-biased (*P*-value ≤ 0.05; fold changes >1.5). After quantification, total protein spots were visualized using silver staining (Fig. 3). All differential protein spots were excised, digested using trypsin, and analysed by liquid chromatography-tandem mass spectrometry (LC-MS/MS). The proteins were identified using the Mascot server against an in-house *S*. *mekongi* transcriptomic dataset (Supplementary Datasets 3 and 4). A total of 88 and 44 proteins were identified as male-biased and female-biased phosphoproteins, respectively (Supplementary Tables 1 and 2). When we compared the 2-DE phosphoproteome results with phosphoproteins predicted using the NetPhos 3.1 program, 108 of 132 (81.8%) identified proteins contained at least one phosphorylation site over the significant threshold (score >0.99). Therefore, all MS-identified phosphoproteins were a subset of the predicted *S*. *mekongi* phosphoproteome.

**Figure 4:**
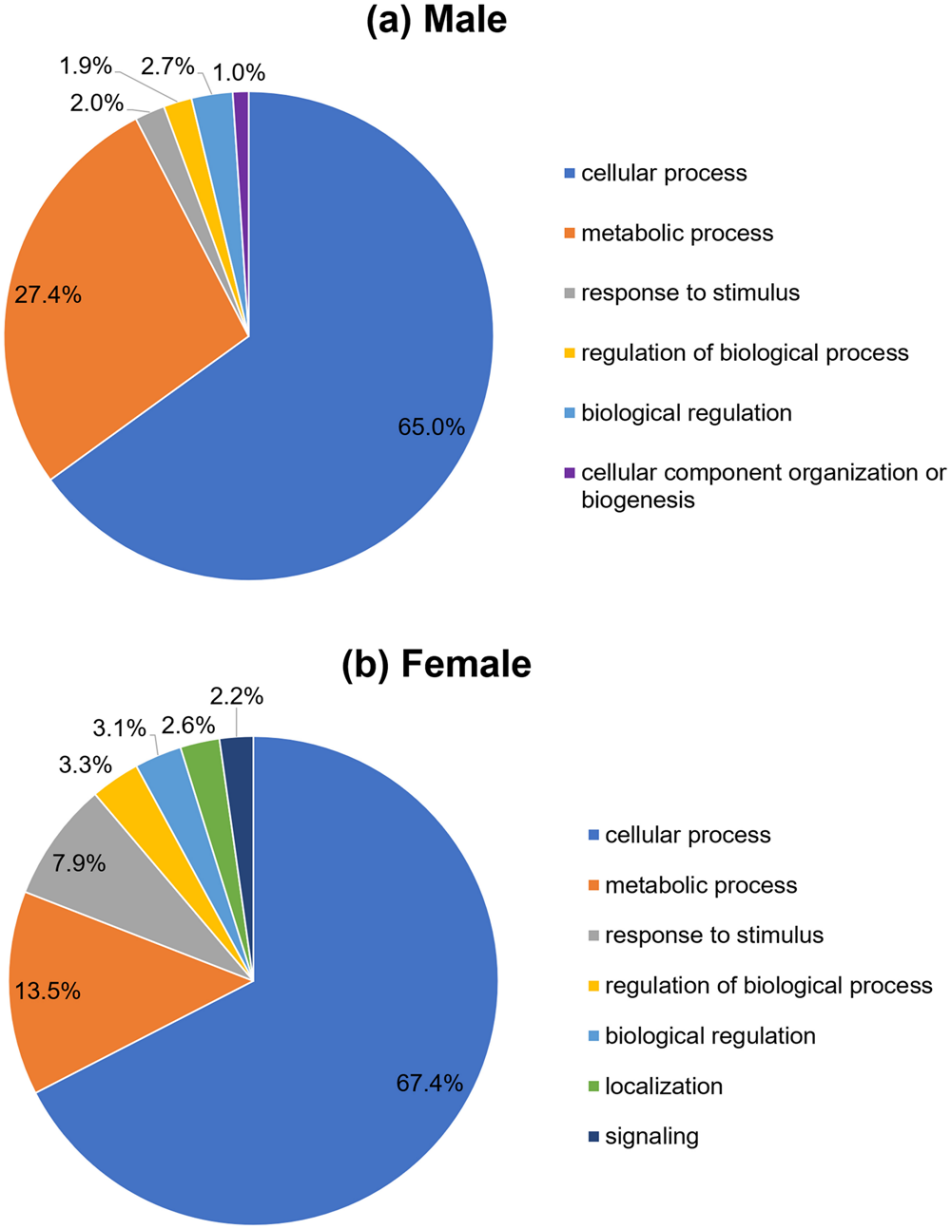
Gene ontology classification according to biological process term of up-regulated phosphoproteins in *S*. *mekongi* male (**a**) and female (**b**) adult worms.

Gene ontology classification using the “biological process” term was performed on the differential phosphoproteins in worms of both sexes, as shown in Fig. 4. “Cellular process” was the major protein class of *S*. *mekongi* male-biased (65.0%) and female-biased (67.4%) phosphoproteins. Phosphorylated actin filament, muscle contraction, and microtubule were included in the “cellular process” class. Typically, motor activity and muscle regulation are activated by phosphorylation. Thus, an increase in phosphorylation of filament and microtubule proteins may be important for schistosome mobility. The phosphoproteins in the “metabolic process” class were almost 2-fold higher in male worms (27.4%) than in female worms (13.5%). In males, the example proteins in this class were aldolase, glyceraldehyde 3-phosphate dehydrogenase, aldehyde dehydrogenase 1B1, and enolase. The “metabolic process” class included proteins in catabolism, a cellular activity that converts complex substances into smaller products. A phosphorylated pathway of catabolism may be essential for energy production in adult males. *S*. *mekongi* female-biased phosphoproteins in the “metabolic process” class included calumenin, enolase, peptide elongation factor 1-beta, and UTP-glucose-1-phosphate uridylyl transferase. These proteins were involved in biosynthesis, which may be associated with embryonic development. Thirty-four of 108 differential phosphoproteins identified in this study may have roles in the reproductive system, as shown in Table 1. In males, they are involved in the reproductive tract, male gonad, germinal cell, epididymis, and sperm; in females, they are involved in gonad and ovary.

**Table 1:** Functions of phosphoproteins in reproductive system.

Function	Protein	Description
Reproductive tract	Myosin heavy chain	Detected within the walls of the reproductive and alimentary tracts of male and female *Brugia malayi* worms^22^.
	Elongation factor	Linked signals from the reproductive system^73^.
Gonad	Proteasome subunit alpha type-3	Involved in proteostasis maintenance in the gonads plausibly facilitates proteome stability across generations^74^.
	Tubulin beta-4B chain	Presented in the reproductive organs and other important organs of *Opisthorchis viverrini*^75^.
	Retinal dehydrogenase 1	Involved in reproduction and development^76^.
	Paramyosin	Detected within the walls of the reproductive and alimentary tracts of male and female *Brugia malayi* worms^22^.
	Glutathione S-transferase M1	Expressed in all part of epididymides and played role as enzymatic barrier protecting for sperm which against the toxic condition of electrophilic compounds in another of reproductive organs^26^.
Germinal cell	Heat shock protein 60	Detected in human germinal cells^39^.
Epididymides	GPI anchored surface glycoprotein	One of originating proteins in epididymides which is present on plasma membrane of sperm^32,33^.
	V-type proton ATPase subunit B	Expressed in epididymis^77^.
Sperm	Dihydrolipoamide dehydrogenase	Involved in capacitation of hamster spermatozoa^78^.
	14-3-3 protein	Essential for normal spermatogenesis by interacting with vimentin in Sertoli cells^36^.
	Aldehyde dehydrogenase X	Maintained stallion sperm motility^42^.
	Heat shock protein 70	Contained in spermatocyte^39^.
	Heat shock cognate 71 kDa	Found in mouse spermatogenic cells^40^.
	F-actin-capping protein subunit beta	Facilitated capacitation and acrosome reaction in mammalian sperm^79^.
	Enolase	Involved in sperm structural and male fertility^43^.
	Pyruvate kinase	Localized at the fibrous sheath and the acrosome of spermatozoa^45^.
	Aldolase	Localized at sperm subcellular components^80^.
	Glyceraldehyde 3-phosphate dehydrogenase	Bound to the fibrous sheath, a cytoskeletal structure that extends most of the length of the sperm flagellum^47^.
	Triosephosphate isomerase	Played an important role as a critical source of energy for motility in mouse sperm^81,82^.
	Annexin	Bound to plasma membranes of human spermatozoa^49^.
	Malate dehydrogenase	Participate in capacitation and acrosome reaction of boar spermatozoa^50^.
	Lactate dehydrogenase	Provided energy metabolism in mouse sperm^51^.
	Phosphoglycerate kinase	Essential for sperm function^52^.
Ovary	Protein disulfide-isomerase	Presented in ovaries of the giant tiger shrimp^53^.
	Titin	Associated with antral follicle counts^54^.
	Actin	Involved in mammalian oocyte meiosis^55^.
Hormone	Flotillin 1	Played role as estrogen responsive gene^59^.
	Calcium-binding EF-hand	Appeared to be under the control of the steroid hormones oestrogen and progesterone in the female reproductive system^58^.
Others	UTP–glucose-1-phosphate uridylyltrans- ferase	Involved in reproductive phases in *Arabidopsis thaliana*^83^.
	Activator of 90 kDa heat shock protein ATPase	Related to fertility in drosophila^39^.
	Ribosomal RNA-processing protein 8	Essential for Reproduction in *Arabidopsis thaliana*^84^.
	Adenylate kinase	Involved in diabetic pregnancy^85^.

### Immunohistochemistry

**Figure 5:**
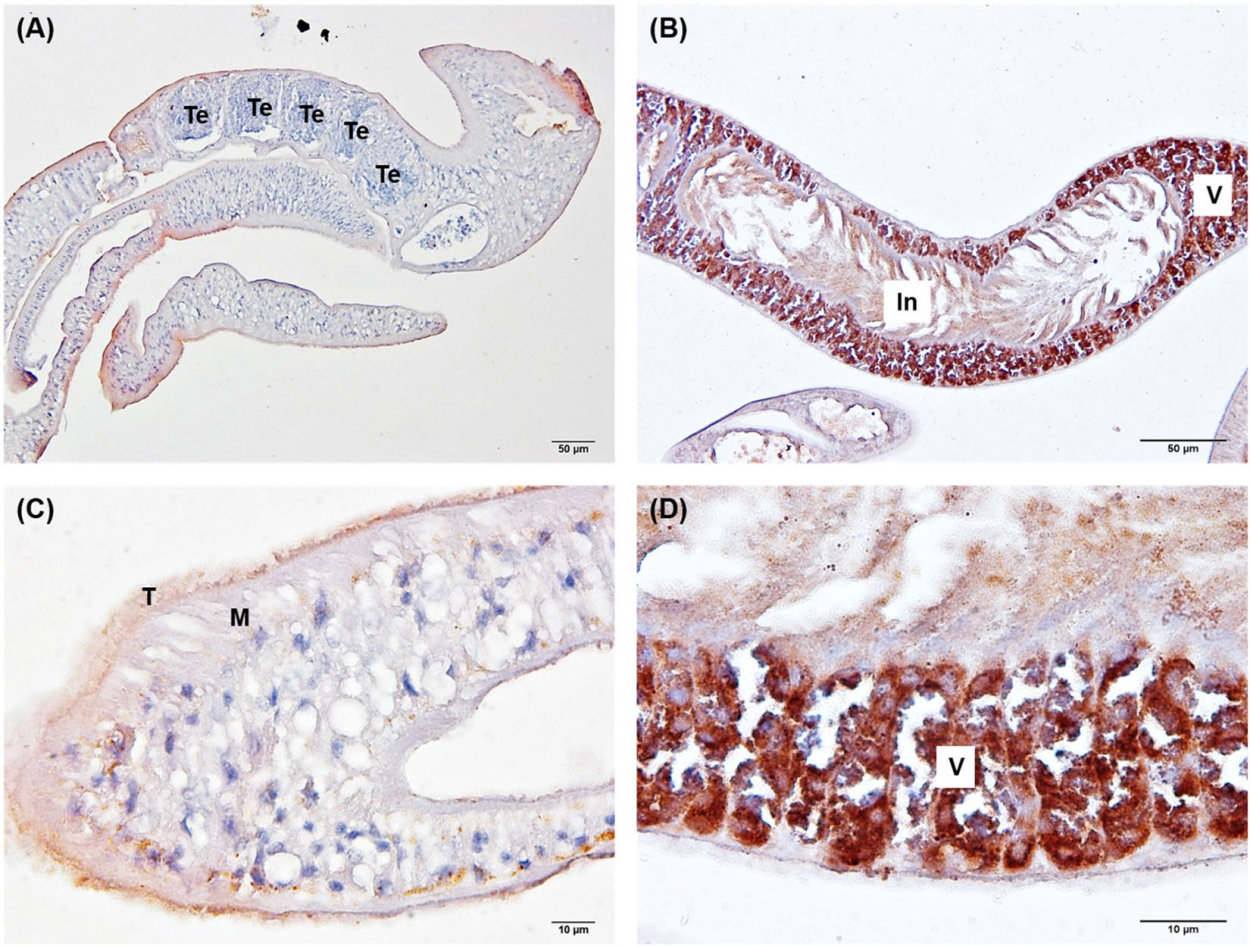
Immunoperoxidase staining of *S*. *mekogi* male and female adult worms. Anti-phosphoserine was used as primary antibody for visualizing phosphoserine in male and female worms. (**A**) Detection of phosphoserine in *S*. *mekongi* male by IHC, (**B**) Detection of phosphoserine in *S*. *mekongi* female by IHC, (**C**) Enlarged IHC image of male worm, (**D**) Enlarged IHC image of female worm.

To reveal the distribution of phosphoproteins on *S*. *mekongi* adult worms, we applied an immunohistochemistry technique (IHC) to both male and female *S*. *mekongi* worms. Phosphorylation sites in eukaryotic organisms occur mainly on serine, threonine, and tyrosine residues at a ratio of 1800:200:1^16^. Here, we used anti-phosphoserine to localize phosphoproteins on *S*. *mekongi* adult worm tissue (Fig. 5). The results showed that tegument (T) and muscle (M) of male worms were phosphorylated, especially on the worm surface; a few signals showed staining of the male reproductive organ, the testis (Te). In contrast, female worms showed a high degree of phosphorylation on the organ for the vitelline gland (V) and the gastrointestinal tract (In).

## Discussion

In this study, we applied computational and experimental approaches to identify phosphoproteins. According to NetPhos 3.1 prediction, the default threshold for analysis is generally 0.5. However, we designed this study to identify phosphorylation sites with very high stringency; thus, we set the threshold for NetPhos 3.1 analysis at 0.99 for phosphoprotein identification. It has been estimated that approximately 50% of a eukaryotic proteome undergoes phosphorylation. In the current study 74% of the *S*. *mekongi* transcriptome was predicted to be phosphorylated. However, because schistosomes have fewer protein kinases (223)^17^ than humans (518)^18^, the biological phosphorylation events between human and schistosomes could be expected to be dissimilar. There is also the possibility that predicted sites may not be accessible to a protein kinase due to subcellular compartmentalisation and 3-D protein structure. Thus, the predicted phosphoproteome obtained using NetPhos is likely to have generated false positive phosphorylation sites.

To quantify the *S*. *mekongi* phosphoproteome, the phosphoproteins of male and female *S*. *mekongi* adult worms were compared using traditional 2-DE. All differential phosphoproteins were classified by gene ontology (Fig. 4). Cellular and metabolic processes were the major differential phosphoprotein classes in *S*. *mekongi* of both sexes. Our findings were consistent with the phosphoproteome gene ontology analysis of *S*. *japonicum* in several stages such as schistosomula, adult male, and adult female^19^. In the cellular process of *S*. *mekongi* male worm, motor activity and muscle regulation were activated by phosphorylation. The increase of phosphorylation on filament and microtubule proteins may be important for schistosome mobility. Additionally, glutathione S-transferase (GST) and peroxiredoxin, classified in the cellular process, were male-biased phosphoproteins. Because the *S*. *mekongi* female resides in the gynaecophoral canal of the male, it may explore the surrounding environment less. The highly phosphorylated antioxidant proteins in males might contribute to detoxification of the host immune response. We detected a greater number of upregulated phosphoproteins in the metabolic process class in makes than in females for example, aldolase, glyceraldehyde 3-phosphate dehydrogenase, aldehyde dehydrogenase 1B1, and enolase. The *S*. *mekongi* male carries the female and moves from the liver against the blood flow into the inferior mesenteric veins^20^. Therefore, highly phosphorylated proteins in the metabolic process class may be associated with production of energy required for male mobility. In *S*. *japonicum*, glycogen is degraded for muscle contraction or tegumental membrane repair, which are more prevalent in adult males^21^. Phosphorylation is involved not only in general behaviours of schistosomes but also in their mating systems. Table 1 summarizes information on gender-biased phosphoproteins of *S*. *mekongi* involved in the reproductive system. Myosin heavy chain and paramyosin have been found within the reproductive tracts of male and female *Brugia malayi* worms^22^. They may function as calcium-dependent regulator of muscle contraction in the genitalia. In *S*. *mansoni*, myosin heavy chain binds strongly to PZQ and is phosphorylated *in vivo* upon exposure to PZQ, as confirmed by immunoprecipitation^23^. Whereas, *Schistosoma* paramyosin has been studied as a promising vaccine candidate for both *S*. *mansoni* and *S*. *japonicum*^24^. Immunization with native *S*. *mansoni* paramyosin induced 39% protection in mice^24^, and vaccination of mice with *S*. *japonicum* paramyosin induced 86% protection^25^. Therefore, myosin heavy chain and paramyosin may be a good target for a multi-species vaccine against schistosomiasis. GST exhibits antioxidant activity and is expressed in bull and boar epididymis. It plays a role in sperm protection against toxic conditions in female immune system^26^. In human, GST is hyper-phosphorylated and enzymatically more active under oxidative stress^27^. It has been studied as a vaccine candidate against schistosomiasis in several models, including nonhuman primates^28–30^. A 50% reduction in the number of eggs of *S*. *haematobium* was demonstrated in GST-immunized patas monkeys^28^. *S*. *mansoni* GST immunization reduces total worm burden and liver egg counts in mice^29^. In addition, mean faecal egg counts of *S*. *japonicum* were reported to be significantly reduced in GST-vaccinated sheep^31^. Thus, GST is a potential candidate for development of a broad-spectrum *Schistosoma* vaccine. Glycophosphatidylinositol (GPI)-anchored surface glycoprotein is present in mouse sperm plasma membrane^32,33^. This phosphoprotein may play a role in movement of the *S*. *mekongi* adult worm and its sperm. In *S*. *mansoni*, GPI-anchored surface glycoprotein has been evaluated as a potential tegument antigen for vaccine development. Mice immunized with *S*. *mansoni* GPI-anchored protein showed a 42% reduction in worm burden and 45% reduction in eggs per gram of hepatic tissue^34^. Dihydrolipoamide dehydrogenase can oxidize dihydrolipoamide to lipoamide. In mouse, phosphorylation of tyrosine residues in dihydrolipoamide dehydrogenase is important for sperm motility^35^. Therefore, phosphorylated dihydrolipoamide dehydrogenase may participate in sperm movement of male schistosomes. The 14-3-3 protein is essential for human spermatogenesis^36^. Moreover, a number of additional phosphorylation sites have been reported in mammalian and yeast isoforms of the 14-3-3 protein^37^. The 14-3-3 protein has also been studied as a candidate vaccine against schistosomiasis^38^. Heat shock proteins (Hsp)60, 70, 71, and 90 are present on spermatocytes^39,40^. Importantly, Hsp70 of schistosome contributes to cercaria-schistosomulum transformation and is associated with cercarial host invasion^41^. Because heat shock protein is involved in the early stage after infection, it is a fascinating target by which to block invasion by schistosomes. Aldehyde dehydrogenase, enolase, pyruvate kinase and glyceraldehyde 3-phosphate dehydrogenase (GAPD) participate in carbohydrate catabolism. Aldehyde dehydrogenase has a demonstrated function in maintaining sperm motility Gibb^42^. However, there is little information on this protein in schistosomes. While, enolase has been found in mouse sperm^43^ and a host-interactive tegumental enzyme in *S*. *mansoni*. In addition, enolase can bind plasminogen and promote its activation, which facilitates the degradation of fibrin polymers. Therefore, schistosomes are able to survive in the human blood system without inducing blood clots^44^. Enolase may be involved in *S*. *mekongi* male fertility and survival. Pyruvate kinase is localized on the fibrous sheath and acrosome of human spermatozoa^45^. In schistosomes, pyruvate kinase activity is inhibited by the antimalarial drug artemether^46^. GAPD binds to the fibrous sheath, a cytoskeletal structure that extends length of the sperm flagellum^47^. In *S*. *mansoni*, this protein has been reported as a vaccine candidate because it is localized on the surface membrane of lung-stage schistosomula^48^. GAPD is expressed only during mouse spermatogenesis and, like its human orthologue, it has roles in sperm motility and movement^47^. As reported in other researches, these proteins in carbohydrate catabolism may play an important role on sperm movement of *S*. *mekongi*. Triosephosphate isomerase, lactate dehydrogenase, malate dehydrogenase, and phosphoglycerate kinase are essential for efficient energy production. Interestingly, these enzymes participated in energy supply for motility of mouse, boar, and human spermatozoa^49–52^. As in these other organisms, these proteins may be involved in energy production, which is important for *S*. *mekongi* sperm movement. Protein disulfide-isomerase, titin, and actin are associated with ovulation in mammals and shrimp^53–55^. In addition, protein disulfide-isomerase and actin have been found on *S*. *mansoni* eggshell^56^. Inhibitors of actin tyrosine phosphorylation could interfere with polymerization of actin during capacitation of buffalo spermatozoa^57^. Thus, these structural proteins may be associated with *S*. *mekongi* egg and sperm production. Flotillin 1 and calcium-binding EF-hand control the steroid hormone oestrogen in human females^58,59^, and phosphorylation of these proteins is involved in signal transduction^60,61^. Thus, phosphorylated flotillin 1 and calcium-binding EF-hand may regulate *S*. *mekongi* reproductive hormones. We hypothesized that phosphorylation may play roles on reproductive system of *S*. *mekongi*.

In this study, eight phosphopeptides could be detected from MS results (Supplementary Table 3). T14, S102, T113, S442 and Y10 were identified as phosphorylation sites of ribosomal RNA-processing protein 8, E3 ubiquitin-protein ligase RNF, heat shock protein 60, Smp_163000 and Smp_169660, respectively. In collision-induced dissociation (CID), phosphopeptides typically results in a neutral loss of the phosphate group^62^. Therefore, assignment of phosphorylation site could be predicted to the presence of possible phosphorylated residues in a peptide. Some peptides containing several serine, threonine, and tyrosine residues in their sequences are unable to indicate the exact phosphorylation sites such as MS3_06728, Smp_060620 and pantothenate kinase 4. According to functional domain prediction using pfam server, E3 ubiquitin-protein ligase RNF and heat shock protein 60 were phosphorylated on their functional domains as shown in Supplementary Fig. 3. E3 ubiquitin-protein ligase RNF is an ER-associated degradation machinery. Malfunction of E3 ubiquitin-protein ligase RNF during early stages of gonad development led to abnormalities in germline development^63^. Phosphorylation of the E3 ubiquitin ligase RNF by a kinase is required for its activity^64^. Therefore, this protein may play an important role in *S*. *mekongi* reproductive organ development. Heat shock protein 60 presented on human sperm surface^39,40,65^. Activity of heat shock protein 60 has been reported to regulate by phosphorylation^66,67^. This protein undergoes tyrosine phosphorylation and become exposed on the cell surface during the capacitation of mouse sperm^68^. Since heat shock protein 60 may involve in sperm production of *S*. *mekongi*, inhibiting phosphorylation of this protein may lead to male infertility. As described above, phosphorylated E3 ubiquitin ligase RNF and heat shock protein 60 are possible to be drug and vaccine targets for schistosomiasis. The antischistosomal drug development could be accomplished through phosphorylation inhibitors.

To elucidate the dominant phosphorylated organs of *S*. *mekongi*, anti-phosphoserine was used for immunohistochemistry. Tegument and muscle of male worm were phosphorylated. The IHC corresponded to the phosphoproteomic results that phosphorylated actin filament, muscle contraction and microtubule related proteins were up-regulated in *S*. *mekongi* adult male such as titin, paramyosin and myosin. In *S*. *mekongi* female, organ for egg shell production, vitelline cells and gastro-intestinal tract were highly phosphorylated. The results also correlated with the phosphoproteomic finding that several up-regulated phosphoproteins in female adult worm were participated in egg production, embryogenesis nutrient consumption.

In conclusion, phosphoproteomics approaches could identify several drug and vaccine target candidates, which may contribute to an alternative schistosomicide development. Moreover, the research finding provided more insight to the *S*. *mekongi* molecular biology.

## Methods

### Phosphorylation site prediction

*S*. *mekongi* male and female transcriptomic datasets^69^ were predicted their phosphorylation sites using NetPhos 3.1 server (http://www.cbs.dtu.dk/services/NetPhos/). The significant positive sequences at threshold greater than 0.99 with at least one phosphorylation site were reported as a phosphoprotein.

### *S*. *mekongi* adult worm preparation

All procedures performed on animals in this study were conducted following the ethical principles and guidelines for the use of animals at the National Research Council of Thailand (NRCT) and with permission from the Faculty of Tropical Medicine Animal Care and Use Committee (FTM-ACUC), Mahidol University. The approval number was FTM-ACUC No. 003/2017. Briefly, *N*. *aperta* shaded *S*. *mekongi* cercaria out after 4 weeks of infection. Afterwards, each ICR mouse (*Mus musculus*) was exposed to 25–30 cercariae at abdomen area by hairpin loop. The infected mice were maintained for 8 weeks. The adult worm collection was done by vascular perfusion using 0.85% normal saline solution and stored in −80 °C until used.

### Protein lysate preparation

Ten *S*. *mekongi* adult male and female worms were separately snap frozen in liquid nitrogen and ground with a mortar and pestle. A 300 µl of lysis buffer containing 8M urea (OmniPur®, Germany), 2M thiourea (Merck, Germany), 4% CHAPS (Thermo Scientific, USA), and 50 mM Dithiothreitol (DTT) (OmniPur®, Germany) was added to each sample. The worm lysates were further ultrasonicated on ice. Cell debris was removed by centrifugation at 12,000 × g for 5 minutes at 4 °C. The supernatants were collected and performed protein precipitation using 2-D clean-up kit followed the manufactural protocol (GE Healthcare, Germany). Protein concentration was determined using Quick Start Bradford protein assay (Bio-Rad, USA).

### Two-dimensional gel electrophoresis (2-DE)

The 2-DE was performed following the previous publication with some modification^70^. In detail, proteins were separated according to their isoelectric point (pI) using a 7 cm immobilized pH gradient (IPG) strip (pH 3–10, NL) (GE Healthcare, Germany). The strip was rehydrated overnight in rehydration buffer including 5 M Urea, 2 M Thiourea, 50 mM DTT, 4% CHAPS and IPG buffer. Isoelectric focusing was performed using an Ettan™ IPGphor™ 3 (GE Healthcare, Germany). The strips were equilibrated in 50 mM DTT in equilibration buffer containing 6 M Urea, 75 mM Tris-HCl, 70 mM SDS, 30% Glycerol for 15 minutes and 125 mM iodoacetamide in equilibration buffer (Thermo Scientific, USA) for 15 minutes. The strip was placed and separated by 12% acrylamide gel (Bio-Rad, USA). All 2-DEs were stained by Pro-Q® Diamond Phosphoprotein gel stain (Thermo Scientific, USA) and silver stain. Three biological replicates were performed for each sample.

The phosphoproteins were visualized by a Typhoon Trio scanner (GE Healthcare, USA). The instrument was controlled by Typhoon scanner control version 5.0 (GE Healthcare, USA). Gel images were analyzed and phosphoproteins quantified by ImageMaster 2D Platinum 7.0 software (GE Healthcare, USA). Spots quantification were determined based on the percentage volume. Protein spots with at least a 1.5-fold difference and ANOVA significance at p-value ≤ 0.05 were selected for MS analysis. Silver staining was used for spot detection and the spots of interest were cut for further in-gel digestion and MS analysis.

### In-gel tryptic digestion

All gel pieces were incubated with 30 mM potassium ferricyanide (K_3_Fe(CN)_6_) (Merck, USA) and 100 mM sodium thiosulfate (Merck USA) solution for destaining. Proteins were reduced by 4 mM DTT at 60 °C for 15 minutes and alkylated by 250 mM iodoacetamide at room temperature for 30 minutes. The gel pieces were dehydrated by 100% acetonitrile (ACN) (Thermo Scientific, USA) and digested by trypsin (Sigma-aldrich, USA, T6567) overnight at 37 °C. The peptides were extracted by adding 100% ACN and incubated for 20 minutes. The samples were stored at −20 °C prior to mass spectrometric analysis.

### Mass spectrometry analysis

Peptide mixture was analysed by an Ultimate® 3000 Nano-LC systems (Thermo Scientific, USA) coupled with a microTOF-Q II (Bruker, Germany). The acquisition was controlled by HyStar™ version 3.2 (Bruker, Germany). MS and MS/MS spectra covered the mass range of m/z 400–2000 and m/z 50–1500, respectively. The raw data format (.d) files were processed and converted to mascot generics files (.mgf) using Compass DataAnalysis™ software version 3.4 (Bruker, Germany) and submitted for database searches using Mascot Daemon software (Matrix Science, USA) against in-house transcriptomics database. Miss cleavage was allowed at one. Variable modifications were set as carbamidomethyl (C), oxidation (M), phospho (ST), and phospho (Y), MS peptide tolerance was 0.8 Da and MS/MS tolerance was 0.8 Da. Differential phosphoproteins were classified by gene ontology using Blast2Go software. Protein domains were predicted by Pfam 32.0 (September 2018, 17929 entries). Swiss model server was used for three-dimensional (3D) structure modeling^71^. The template was selected by a sequence with the highest percentage identity. The.pdb file of modeled protein structures were downloaded and analyzed by Visual Molecular Dynamics software^72^.

### Immunohistochemistry

Adult male and female worms were fixed overnight at 4 °C in 10% neutral buffer formalin. The worms were dehydrated through an ethanol series, therefore infiltrated and embedded in graded paraffin. The embedded worms were cut into 4 µm thick and placed on pre-coated immunohistochemistry slides. Heat-induced antigen retrieval with citrate buffer (pH 6) was used for enhancing tissue antigenicity. EnVision FLEX/HRP (K8002; DAKO, Denmark) and EnVision G/2 System/AP (K5355-11; DAKO, Denmark) kits were used for peroxidase and alkaline phosphatase staining systems, respectively. Subsequence to non-specific binding and endogenous peroxidase blocks, anti-phosphoserine (Merck, USA, AB1603) was applied to the tissue at 1:100 dilution. Regard to the staining systems, the tissue was then incubated in secondary antibody conjugation kits, visualized by either 3, 3-diaminobenzidine (DAB) or liquid permanent red (LPR), and counter stained by hematoxylin. Immunolocalization was examined under a light microscope (BX51, Olympus, Japan) with digital camera (DP20, Olympus, Japan).

## Supplementary information

Supplementary information

Supplementary dataset1

Supplementary dataset2

Supplementary dataset3

Supplementary dataset4
