# Supplementary material for: Phosphoproteomics analysis of male and female Schistosoma mekongi adult worms
Source: Sci Rep. 2019 Jul 10;9:10012. doi: 10.1038/s41598-019-46456-6 (PMC6620315; doi:10.1038/s41598-019-46456-6)
Supplement: Supplementary file 1 — Supplementary information [file 41598_2019_46456_MOESM1_ESM.pdf]

Phosphoproteomics analysis of male and female *Schistosoma mekongi* adult worms

Nattapon Simanon[1], Poom Adisakwattana[2], Tipparat Thiangtrongjit[1], Yanin Limpanont[3], Phiraphol Chusongsang[3], Yupa Chusongsang[3], Songtham Anuntakarun[4], Sunchai Payungporn[4], Sumate Ampawong[5], Onrapak Reamtong[1] *

[1] Department of Molecular Tropical Medicine and Genetics, Faculty of Tropical Medicine, Mahidol University, Bangkok, 10400, Thailand

[2] Department of Helminthology, Faculty of Tropical Medicine, Mahidol University, Bangkok, 10400, Thailand

[3] Department of Social and Environmental Medicine, Faculty of Tropical Medicine, Mahidol University, Bangkok, 10400, Thailand

[4] Department of Biochemistry, Faculty of Medicine, Chulalongkorn University, Bangkok, 10330, Thailand

[5] Department of Tropical Pathology, Faculty of Tropical Medicine, Mahidol University, Bangkok, 10400, Thailand

*Correspondence to: Onrapak Reamtong, Department of Molecular Tropical Medicine and Genetics, Faculty of Tropical Medicine, Mahidol University, Bangkok, 10400, Thailand

Email: onrapak.rea@mahidol.ac.th

Tel: 66 (0) 2306-9138

Fax: 66 (0) 2306-9139

Supplementary Figure 1. Modeled structures of E3 ubiquitin-protein ligase and heat shock protein 60. Grey areas indicate functional domains of protein and red structures indicate identified phosphorylated sites

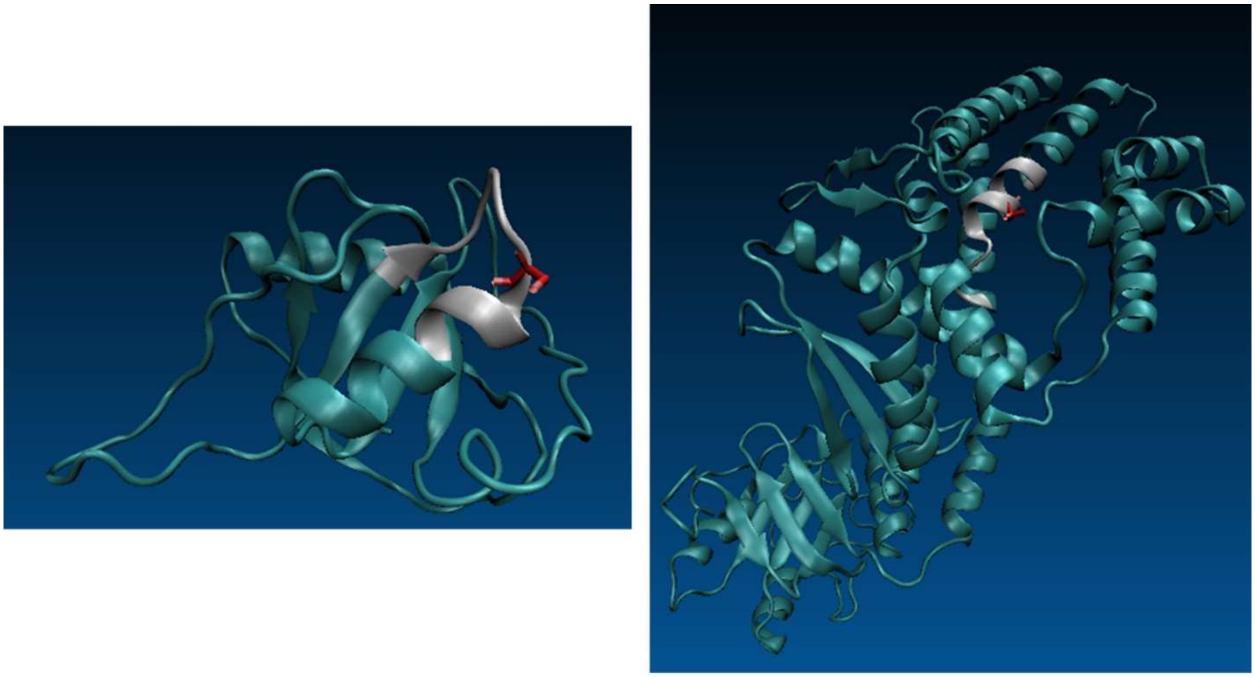

E3 ubiquitin-protein ligase **Heat shock protein 60**

Supplementary Figure 2. Immunogold staining of *S. mekogi* male adult worm. Anti-phosphoserine was used as primary antibody for visualizing phosphoserine in male worms by electron microscopy. Arrows demonstrated the gold particles

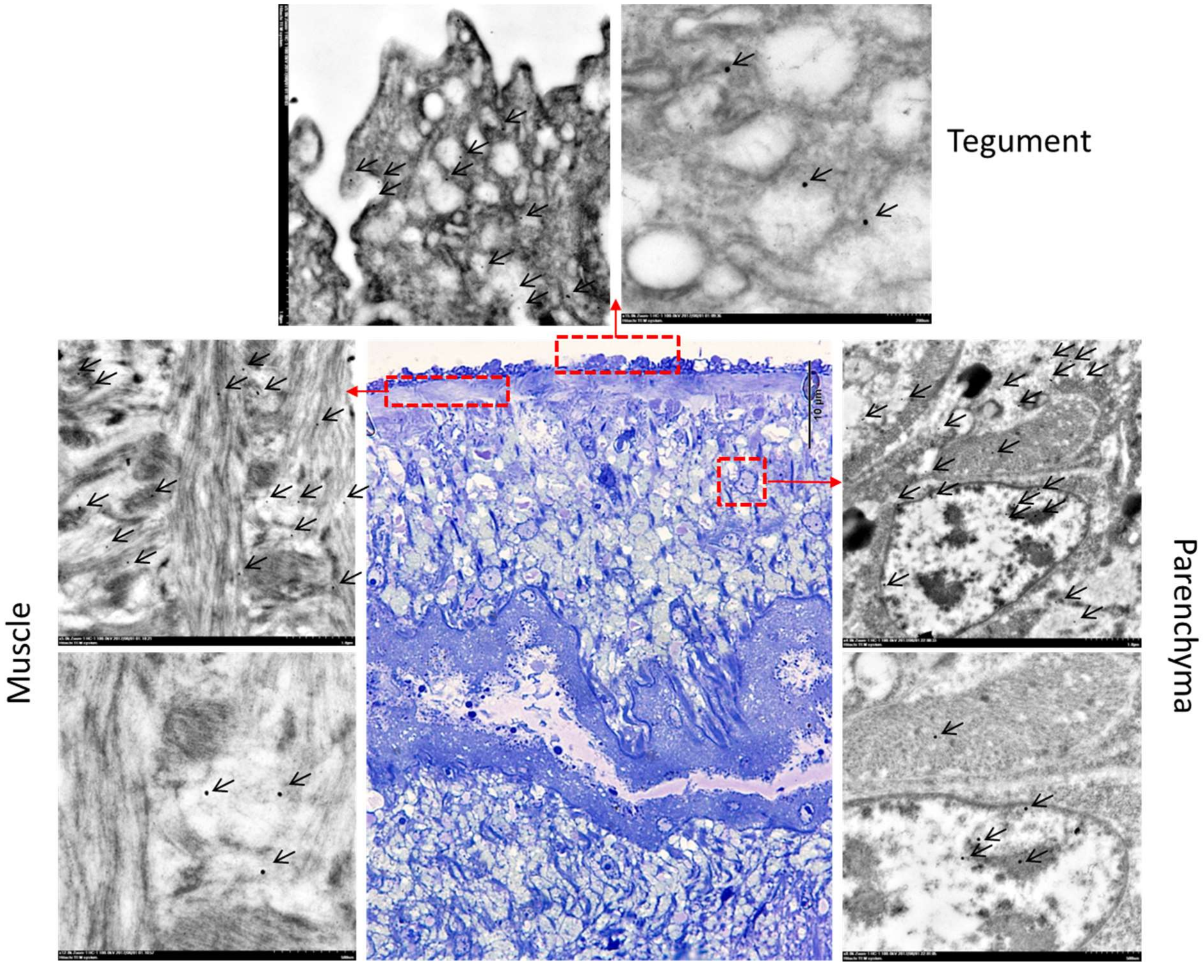

Supplementary Table 1. *S. mekongi* male-biased phosphoproteins identified by mass spectrometry

Spot ID	Entry	Protein	PS	%Cov.	MW	pI	Spot Intensity		Fold Change
							Male	Female	
**1**	U3N6C3	Lethal giant larvae protein	44	0.9	155588	5.02	0.3036	0.0199	4.7255
**2**	Q5DI56	SJCHGC09287 protein	846	16	83169	4.65	0.6118	0.0144	12.3972
	A0A095CAB5	Titin	537	7.8	83233	4.64	0.6118	0.0144	12.3972
**3**	Q05870	Paramyosin	498	19.2	100480	5.3	1.6134	0.0622	10.116
	Q8MPH3	Myosin heavy chain, partial	71	9	38291	4.94	1.6134	0.0622	10.116
**4**	Q5D947	SJCHGC00820 protein	1660	32.5	82166	4.98	0.7751	0.0135	20.7944
	C1LEA0	TNF receptor-associated protein 1	253	2	80253	6.26	0.7751	0.0135	20.7944
	O96398	Myosin	78	2.9	92385	5.32	0.7751	0.0135	20.7944
	Q5DH53	Unknown	51	2.8	40453	5.83	0.7751	0.0135	20.7944
**7**	C1LIA3	Dihydrolipoamide dehydrogenase	83	7.4	52986	6.63	0.0432	0.0042	2.8121
**8**	Q5C1H0	SJCHGC05356 protein, partial	156	19.7	15051	6.73	0.1177	0.0212	1.7547
	P08418	70,000 mol wt antigen/hsp70 homologue (619 AA), partial	73	8.1	67989	5.4	0.1177	0.0212	1.7547

**10**	C1LFP4	Putative aldehyde dehydrogenase 1B1 precursor	469	20.4	53548	6.06	0.153	0.0444	1.803
**11**	P53471	Actin	495	31.4	41714	5.3	0.2888	0.1081	2.0494
	Q5DH23	SJCHGC09129 protein	186	8.9	62101	9.51	0.2888	0.1081	2.0494
**12**	P53471	Actin	1097	44.9	41714	5.3	0.2893	0.0733	2.0974
**13**	C7TZI6	Heat shock protein 60	1404	48.1	50572	6.88	0.3165	0.0453	4.2215
	O45039	HSP70, partial	40	2.9	55971	5.38	0.3165	0.0453	4.2215
	A0A095BWG5	Hypothetical protein MS3_01515	40	0.8	127954	9.02	0.3165	0.0453	4.2215
**14**	Q02456	Myosin heavy chain	46	0.5	222243	5.55	0.8487	0.3391	2.0629
	Q5DC37	SJCHGC02147 protein	41	1.7	53526	5.63	0.8487	0.3391	2.0629
	Q5BYY8	SJCHGC04813 protein	39	4.3	24581	5.04	0.8487	0.3391	2.0629
**15**	Q5DI35	SJCHGC00471 protein	168	12.2	47309	6.18	0.1502	0.0106	5.4785
	Q27877	Enolase	73	8.8	46965	6.18	0.1502	0.0106	5.4785
	Q5DAY9	SJCHGC06539 protein	43	3.2	26413	8.13	0.1502	0.0106	5.4785
**17**	C1LVD4	Tubulin beta-2C chain	1802	53.5	49599	4.8	0.8487	0.2156	2.055
	Q5C0B0	SJCHGC09060 protein	152	9.6	40124	4.75	0.8487	0.2156	2.055

	Q5D9S4	SJCHGC09424 protein	86	6	55675	5.85	0.8487	0.2156	2.055
	G4VLW2	Actin	40	2.7	41705	5.3	0.8487	0.2156	2.055
**20**	Q5DDV5	Enolase	254	22.8	47221	6.18	0.175	0.0401	1.5606
**26**	P53471	Actin	361	21	41714	5.3	1.5986	0.7205	1.5608
	Q5DGG9	Unknown	185	11.6	37932	5.84	1.5986	0.7205	1.5608
**27**	P53471	Actin	314	22.1	41714	5.3	2.7697	0.6464	3.2995
	Q5DGG9	Unknown	50	4.4	37932	5.84	2.7697	0.6464	3.2995
**28**	P53471	Actin	705	41.2	41714	5.3	2.3339	0.6875	2.7664
	Q5DGG9	Unknown	105	8.2	37932	5.84	2.3339	0.6875	2.7664
	Q5DDV5	Enolase	49	2.3	47221	6.18	2.3339	0.6875	2.7664
	C1LED2	Calcium-binding EF-hand,domain-containing protein	41	15.5	12132	4.65	2.3339	0.6875	2.7664
**29**	Q5DGG9	Unknown	367	16.7	37932	5.84	0.7183	0.178	3.3821
	C1LED2	Calcium-binding EF-hand,domain-containing protein	256	15.5	12132	4.65	0.7183	0.178	3.3821
	P53471	Actin	174	28.2	41714	5.3	0.7183	0.178	3.3821

	Q5BWA0	SJCHGC05635 protein	100	4.9	29135	8.84	0.7183	0.178	3.3821
	A0A095AR71	Hypothetical protein MS3_05150	41	2.3	40426	8.42	0.7183	0.178	3.3821
**30**	Q5D9E1	SJCHGC06074 protein	76	2.6	39097	6.06	0.1548	0.0681	1.6025
**31**	Q964P2	Elongation factor 1-a	125	3.2	38350	6.87	0.0558	0.0143	1.5548
**32**	B3W666	Unknown	1785	47.5	33006	4.63	0.3324	0.0705	1.5758
	Q5D9R3	SJCHGC09402 protein	73	3.8	34604	4.7	0.3324	0.0705	1.5758
	G4VJG0	Hypothetical protein Smp_169660	45	1.6	115505	5.7	0.3324	0.0705	1.5758
**33**	Q5DBM0	SJCHGC00845 protein	161	12.4	39648	4.95	0.2034	0.0404	1.6645
	C1L7Y4	Annexin A13 (Annexin XIII)	144	10.2	39584	5.1	0.2034	0.0404	1.6645
	A0A094ZR35	Hypothetical protein MS3_04935	53	2	43895	9.11	0.2034	0.0404	1.6645
	G4V6H8	Hypothetical protein Smp_060620	40	6	20642	4.76	0.2034	0.0404	1.6645
**38**	P53471	Actin	212	17.3	41714	5.3	0.2029	0.0461	1.5198
	A0A095ATE6	F-actin-capping protein subunit beta	139	13	31338	5.33	0.2029	0.0461	1.5198
**42**	Q94745	26kD glutathione S-transferase	361	31.2	25470	6.14	0.1986	0.023	2.9463
**43**	Q94745	26kD glutathione S-transferase	63	22	25470	6.14	0.3166	0.0486	2.7613
**44**	Q94745	26kD glutathione S-transferase	851	44.5	25470	6.14	0.1281	0.031	1.6521

	G4VT71	Hypothetical protein Smp_163000	47	2.3	58216	8.46	0.1281	0.031	1.6521
**46**	C4QFX9	Heat shock protein 70 (hsp70)-4	51	1.3	94404	5.43	0.1062	-	
**47**	C4QFX9	Heat shock protein 70 (hsp70)-4	63	1.3	94404	5.43	0.0803	-	
	C1L4L6	DNA replication factor Cdt1 (Double parked homolog)	40	1.6	64520	9.74	0.0803	-	
**48**	Q5DI56	SJCHGC09287 protein	128	6.7	83169	4.65	0.0851	-	
	Q26607	surface protein, partial	41	0.4	189193	4.93	0.0851	-	
**49**	Q5DAM7	SJCHGC06305 protein	237	16.2	61146	6.48	0.0199	-	
	G4VAH9	Putative pyruvate kinase	95	11.3	54463	6.79	0.0199	-	
**50**	C1L4L6	DNA replication factor Cdt1 (Double parked homolog)	40	1.6	64520	9.74	0.0733	0.0078	2.4927
**51**	Q8IT62	Trimeric G-protein alpha o subunit	256	25.6	37542	5.09	0.2905	-	
	G4VLW2	Actin	52	2.7	41705	5.3	0.2905	-	
	Q5DI18	SJCHGC02266 protein	44	3.2	42721	5	0.2905	-	
	G4V8L1	Putative protein transport protein Sec13	41	0.8	96600	7.56	0.2905	-	
**52**	P53471	Actin	441	29.5	41714	5.3	0.066	-	

	Q5DGG9	Unknown	67	8.2	37932	5.84	0.066	-
**53**	G4VLW2	Actin	60	2.7	41705	5.3	0.1008	-
	Q5DGT9	SJCHGC05654 protein (Ribosomal RNA-processing protein 8)	39	3	30524	9.47	0.1008	-
**54**	Q86DX2	Fructose bisphosphate aldolase	59	3.8	31720	8.16	0.0368	-
	Q5D9E1	SJCHGC06074 protein	48	5.1	39097	6.06	0.0368	-
	Q964P2	Elongation factor 1-a	46	3.2	38350	6.87	0.0368	-
**55**	Q5DFZ8	SJCHGC00411 protein	341	20.4	39539	6.52	0.3243	-
	G4VJT9	Fructose 1,6 bisphosphate aldolase	266	14.9	39621	7.63	0.3243	-
**56**	C1LB89	Aldolase	484	16.3	39457	6.76	0.347	-
	G4VJT9	Fructose 1,6 bisphosphate aldolase	328	10.7	39621	7.63	0.347	-
	A0A095AHP1	E3 ubiquitin-protein ligase RNF	45	3	52398	7.21	0.347	-
**57**	C1LB89	Aldolase	688	24.8	39457	6.76	0.2566	-
	G4VJT9	Fructose 1,6 bisphosphate aldolase	557	13.5	39621	7.63	0.2566	-
**58**	Q5DHI5	SJCHGC01577 protein	76	7.9	39433	7.56	0.0739	-
	P20287	Glyceraldehyde-3-phosphate dehydrogenase	58	3.3	36355	8.16	0.0739	-

	Q964P2	Elongation factor 1-a, partial	41	3.2	38350	6.87	0.0739	-	
**59**	P20287	Glyceraldehyde-3-phosphate dehydrogenase	45	3.3	36355	8.16	0.0603	-	
	Q5DHI5	SJCHGC01577 protein	45	4.7	39433	7.56	0.0603	-	
**60**	Q86DX2	Fructose bisphosphate aldolase	86	10.8	31720	8.16	0.1795	-	
	C1L4L6	DNA replication factor Cdt1 (Double parked homolog)	40	1.6	64520	9.74	0.1795	-	
**61**	Q76MU5	Glyceraldehyde-3-phosphate dehydrogenase	412	37	24392	8.53	0.1587	-	
	C1LJM5	Malate dehydrogenase	132	14.1	36431	8.7	0.1587	-	
	Q86EU3	Lactate dehydrogenase A	105	7.7	32507	6.65	0.1587	-	
	Q5DHI5	SJCHGC01577 protein	49	4.7	39433	7.56	0.1587	-	
**62**	Q5DD64	SJCHGC00495 protein	51	3.2	35612	6.87	0.1003	-	
**63**	Q5DHV7	SJCHGC01881 protein	201	18.5	29186	4.26	0.1349	0.0165	2.5325
**64**	Q95PM1	Cathepsin B endopeptidase	43	4.9	39082	5.75	0.1511	0.0276	2.1373
**65**	P41759	Phosphoglycerate kinase	45	2.9	44480	6.84	0.1483	-	
**66**	Q5DAH2	SJCHGC05786 protein	101	10.1	21595	5.54	0.0962	-	
	Q94745	26kD glutathione S-transferase	94	11	25470	6.14	0.0962	-	

**67**	Q27775	Triosephosphate isomerase	166	10.3	27602	7.04	0.0804	-
	A0A094ZU44	Hypothetical protein MS3_06728	39	1.1	105862	9.2	0.0804	-
**68**	A0A095A0V3	Peroxiredoxin-2	68	10.5	24890	8.53	0.252	-
	P25824	Adenylate kinase	44	6.1	22330	8.97	0.252	-
**69**	Q03528	22.6kd tegumental associated antigen	57	8.9	22573	6	0.1906	-

Supplementary Table 2. *S. mekongi* female-biased phosphoproteins identified by mass spectrometry

Spot ID	Entry	Protein	PS	%Cov.	MW	pI	Spot Intensity		Fold Change
							Male	Female	
**5**	C7TZG8	Myosin heavy chain, partial	81	7.2	94754	8.49	0.0229	0.1607	4.9165
**6**	B3GUU7	Unknown	541	17.1	71412	5.12	2.0541	4.811	1.8326
	A0A095CGL8	78 kDa glucose-regulated protein	322	11.9	71277	5.21	2.0541	4.811	1.8326
	Q86E38	Heat shock protein 86	125	13.3	30698	6.77	2.0541	4.811	1.8326
	Q5C296	SJCHGC01885 protein	99	2.7	111846	5.26	2.0541	4.811	1.8326
	P06198	Paramyosin	79	2	100326	5.31	2.0541	4.811	1.8326
	C1LEA0	TNF receptor-associated protein 1	62	2	80253	6.26	2.0541	4.811	1.8326
	Q5DH53	Unknown	44	2.8	40453	5.83	2.0541	4.811	1.8326
**9**	C1L502	ER calcistorin	1217	38.4	54388	4.87	0.0722	0.1802	1.7654
	C1LVD4	Tubulin beta-2C chain	220	14.2	49599	4.8	0.0722	0.1802	1.7654
	O45038	HSP70	218	5.4	71442	5.12	0.0722	0.1802	1.7654
**16**	G4VDL3	Putative utp-glucose-1-phosphate uridylyltransferase 2	48	4.5	52695	8.13	0.0353	0.1521	3.1132

**18**	Q5DD74	SJCHGC01701 protein	61	5.5	44598	4.84	0.9834	2.6348	2.2121
	G4VJL0	Putative dynactin subunit	44	2.7	46142	5.03	0.9834	2.6348	2.2121
**19**	Q5DH23	SJCHGC09129 protein	65	1.6	62101	9.51	0.0145	0.0803	1.909
**21**	Q5DHM2	SJCHGC00865 protein	53	2.2	46051	5.71	0.2035	0.4966	1.9812
**22**	G4VHN3	Putative atp synthase beta subunit	1951	45.5	55802	5.22	0.1656	0.5451	3.0211
	Q5D959	SJCHGC06651 protein	58	3.1	31912	5.23	0.1656	0.5451	3.0211
	A0A095AZ59	28S ribosomal protein S28, mitochondrial	40	3.7	31146	7.6	0.1656	0.5451	3.0211
	Q5C0B0	SJCHGC09060 protein, partial	40	3.4	40124	4.75	0.1656	0.5451	3.0211
**23**	P53471	Actin	753	44.7	41714	5.3	0.1178	0.3132	2.0059
**24**	Q5DDV5	Enolase	75	2.8	47221	6.18	0.0578	0.1238	1.5886
**25**	P53471	Actin	789	30.9	41714	5.3	1.2088	3.3589	2.4495
	P06198	Paramyosin	60	2.3	100326	5.31	1.2088	3.3589	2.4495
	Q5DDI7	SJCHGC01371 protein	50	3	41348	5.34	1.2088	3.3589	2.4495
	Q5DBK7	SJCHGC01423 protein	49	5	39023	5.28	1.2088	3.3589	2.4495
	Q86F47	Nuclear distribution gene C homolog	41	2.4	37878	5.33	1.2088	3.3589	2.4495
**34**	A5A6F8	Paramyosin	48	2.3	100368	5.29	0.0684	0.2087	1.7764

	Q86F47	Nuclear distribution gene C homolog	46	2.4	37878	5.33	0.0684	0.2087	1.7764
	G4VLW2	Actin	45	2.7	41705	5.3	0.0684	0.2087	1.7764
**35**	Q86FI4	SJCHGC09235 protein	44	3.8	36449	4.4	0.3801	0.7692	1.5663
**36**	G4VLW2	Actin	60	2.7	41705	5.3	0.2623	1.1171	3.4721
**37**	Q86EU5	Peptide elongation factor 1-beta	45	4.1	23967	4.62	0.7005	3.1582	3.3068
**39**	G4VLW2	Actin	211	8.2	41705	5.3	0.1528	0.3914	2.3752
	Q5DH47	Unknown	90	8.9	13780	4.22	0.1528	0.3914	2.3752
	Q86EE3	Hypothetical protein, AY071024 RE10515p	67	6.3	28256	4.81	0.1528	0.3914	2.3752
	Q5DA69	SJCHGC01759 protein	62	4.7	28821	4.87	0.1528	0.3914	2.3752
	P12795	Heat shock 70 kDa protein	41	4	21832	5.07	0.1528	0.3914	2.3752
**40**	Q5DBQ9	SJCHGC01755 protein	97	15.5	28786	4.9	0.141	0.2747	1.5642
	Q5BXV0	SJCHGC06458 protein	70	14.2	24869	6.29	0.141	0.2747	1.5642
**41**	Q9GUB9	14-3-3 epsilon	169	13.6	28529	5.64	0.0645	0.1818	2.1785
	A0A094ZFU5	Major vault protein	118	15.4	17364	5.49	0.0645	0.1818	2.1785
	Q5DI35	SJCHGC00471 protein	101	5.1	47309	6.18	0.0645	0.1818	2.1785

	Q86F62	Proteasome (prosome, macropain) subunit, alpha type 5	85	8.9	27362	5.22	0.0645	0.1818	2.1785
	Q5C1H0	SJCHGC05356 protein	45	11.7	15051	6.73	0.0645	0.1818	2.1785
	Q5DBB0	SJCHGC02058 protein	42	7.9	18018	5.81	0.0645	0.1818	2.1785
	Q5DAY9	SJCHGC06539 protein	40	3.2	26413	8.13	0.0645	0.1818	2.1785
**45**	Q3KTI0	SJCHGC07036 protein	64	6.3	17243	5.37	0.0808	0.5523	2.3515
	A0A095AK18	Pantothenate kinase 4	45	1.1	94316	6.12	0.0808	0.5523	2.3515

Supplementary Table 3. Phosphorylation sites identified by MS and functional domains predicted by Pfam database

Protein	MS identified phosphopeptide	Phosphorylated residues	Domains	Domain residues
**Ribosomal RNA-processing protein 8**	ILTKPQKK (1 Phospho)	T14	Methyltransferase	37-264
**E3 ubiquitin-protein ligase RNF**	GDCSFVDKALAAER (1 Phospho)	S102	Protease associated	63-154
			Ring finger domain	234-278
**Heat shock protein 60**	LVQDVANNTNEEAGDGTTTATVLAR (1 Phospho)	T113	TCP-1/cpn60 chaperonin family	45-466
**Hypothetical protein Smp_163000**	TCQKPISHLPLK (1 Phospho)	S442	-	-
**Hypothetical protein MS3_06728**	QNSALSGSSR (1 Phospho)	S271/S274/S276/S277	-	-
**Hypothetical protein Smp_060620**	HESSNKTSNIR (2 Phospho)	S246/S245/T250/S251	Pyrimidine 5'-nucleotidase	3-166

**Hypothetical protein Smp_169660**	EMNAAYDNLLADIHKR (1 Phospho)	Y10	-	-
**Pantothenate kinase 4**	SSSKSTSQK (2 Phospho)	S450/S451/S452/S454/S456	Fumble	25-364
			Protein of unknown function DUF89	510-830
